# A Case of Acute Glossitis

**Published:** 1846-07

**Authors:** Thomas Hamilton

**Affiliations:** Thistledale, Cass County, Ga.


*A Case of Acute Glossitis.* By THOMAS HAMILTON, M. D., of Thistledale, Cass County, Ga.—A month ago, a young gentleman whose residence is a little more than thirty miles from this, in the direction of Tennessee, and in a neighbourhood in which several deaths, it is said, have occurred from a disease prevalent there under the popular name of "black tongue," was unfortunate enough while on a visit in my neighborhood to receive a fracture of the thigh bone. The obvious inexpediency of any attempt to carry him home, together with my sympathy for him, moved me to offer him a room at my house, to which he was carried, under the eye of his intelligent physician. Within a few days afterwards, his black man, John, came down from home to wait on him, and this he continued to do without attention to any other business for the last three weeks. John's age is about twenty-five years, he is by nature cheerful and active, has a sound constitution, and was in good health till Tuesday, the 17th of this month. At 11 o'clock in the forenoon of that day, he was heard to speak of a slight smarting sensation in his tongue, affecting mostly its right side, and which he had just then for the first time perceived. Within four hours afterwards, or by three o'clock in the afternoon, this sensation had assumed the character of a constant pain, attended by a sense of heat; and notwithstanding neither his pulse nor skin as yet indicated fever, it was thought proper to prescribe a dose of sulphate of magnesia, which he took without delay. In the course of the afternoon, and before the operation of the salts, headache came on with increased secretion of saliva and a sense of burning in the skin on the right side of his neck, his description of which agreed with that of erysipelas. Neither swelling nor hardness anywhere appeared except in his tongue, which was now perceptibly thickened and somewhat rigid; it was, too, a little more florid than is usual in the healthy state, yet it was moist and but slightly furred. At 7 o'clock of the next morning, his medicine having produced three or four alvine evacuations, his headache was abated and he thought his case in other respects no worse. His pulse, however, had begun to indicate a febrile movement, his articulation was impaired by increased rigidity and tenderness of his tongue, and a long cylinder of viscid saliva hung from his mouth. This body of saliva, which he was unable to discharge by the action of his tongue and lips, he occasionally removed with his hand, but by an active secretion it was immediately reproduced. By one o'clock in the afternoon the case had made fearful progress; the patient's skin was hot and dry, his pulse had acquired a firmness and frequency much above the healthy standard, and the hardness and tenderness having extended from his tongue to all the parts embraced by the rami of the inferior maxillary and the hyoid bones, he was without the power of articulating a word, and at every breath he groaned.

Decisive treatment was now determined on: accordingly, a vein was opened from which the blood was permitted to flow in a large stream until relaxation was denoted by a weakened state of the pulse, anxiety, nausea and sweating. Half an hour afterwards the anxiety and nausea were carried off by the act of vomiting, after which the patient rested quietly. At 5 o'clock, which was the fourth hour after the time of the bloodletting, the case was again examined, when the pulse was found to be recovering its firmness and the skin to be losing its softness. With a view to restrain the circulation steadily and to maintain the softness of the skin, it was deemed expedient to employ a nauseant in aid of the lancet: accordingly the patient was turned upon his back, his head a little elevated, and half a grain of tartar emetic dissolved in a spoonful of water, was, with some difficulty, conveyed into his stomach. Within twenty-five minutes after the administration of this remedy, its effects appeared in a general perspiration, a reduction of the pulse, nausea and restlessness: and the lapse of an hour brought about an effort to vomit, by which these were entirely removed. After this, the state of the pulse and skin continuing good, medication was omitted and the patient, free from pain, rested comfortably through the night. At 7 o'clock of the next morning, he was so far advanced in convalescence as to need no other prescription than that of rest and a gruel diet.

Having read but little, and seen less of diseases for the last ten years, I must leave it to others to decide what affinity, if any, this case bears to the Western epidemic commonly called the "black tongue.'' Yet judging from the rapid progress of the symptoms and the intensity of the inflammation, I may hazard the opinion, that the tendency was to a termination in gangrene, and that nothing was wanting to have made this a case exhibiting a livid appearance of the tongue and contiguous parts, than delay in the use of proper remedies till it should have been too late for them to avert such a termination. Most probably it would have been a case of black tongue, indeed, and one well calculated to discredit the lancet, if the patient in the dread of treatment or hope of amendment, had deferred the use of that remedy until symptoms should have supervened, suggesting the name for his disease.

It is well remembered to have been said by the "greatest and best" in the profession of medicine, that the lancet is the "magnum bonum Dei:" and although this truth will survive the wreck of many an innovation in theory and practice, yet its full benefits can never be realized until it comes to be generally known, that the efficacy of bleeding depends upon its early employment, as cases like this tend to show.—*Southern Med. and Surg. Journ.*

